# Supplementary material for: Oncogenic c-Myc induces replication stress by increasing cohesins chromatin occupancy in a CTCF-dependent manner
Source: Nat Commun. 2024 Feb 21;15:1579. doi: 10.1038/s41467-024-45955-z (PMC10881979; doi:10.1038/s41467-024-45955-z)
Supplement: Supplementary file 1 — Supplementary Information [file 41467_2024_45955_MOESM1_ESM.pdf]

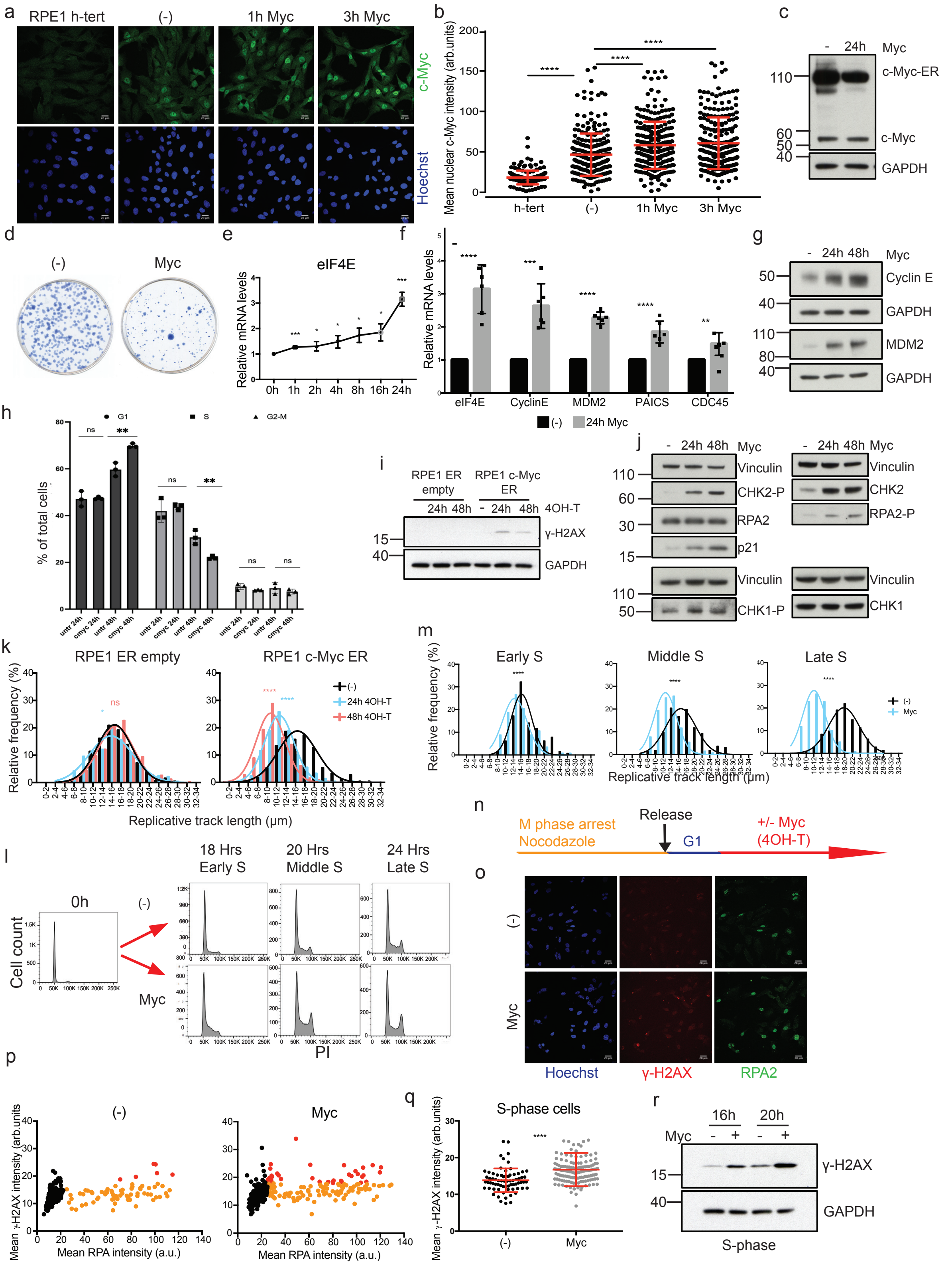

Supplementary figure 1. c-Myc-induced replication stress and DNA damage require G1 phase of the cell cycle.

a) Immunofluorescence staining of c-Myc in RPE1 h-TERT and RPE1 c-Myc-ER cells after addition of 4OH-T for the indicated times.

b) Graph showing c-Myc mean intensity in individual RPE1 h-TERT and RPE1 c-Myc ER cell nuclei after addition of 4OH-T for the indicated times, plotted in the scatter plot. p-value****<0.0001 calculated with two-sided Mann-Whitney test. n=1 experiment. Mean with SD is shown. Source data are provided as a Source Data file

c) Western blot of c-Myc in untreated cells and after 24 hr 4OH-T addition in RPE1 c-Myc ER cells. GAPDH is a loading control. Representative of n=3 experiments.

d) Survival assay of untreated and c-Myc activated RPE1 c-Myc ER cells. Cells were treated with 48 hr with 4OH-T, plated in fresh media and colonies were left to grow for 7 days. Representative of n=4 experiments.

e) Time course of the c-Myc target eIF4E mRNA levels at the indicated time-points after c-Myc activation. p-value***=0.0009 and 0.0006, *=0.02, 0.01, 0.01 calculated with two-sided Student's t-test. n=4 experiments. Mean with SD is shown. Source data are provided as a Source Data file

f) mRNA levels of different c-Myc targets at 24 hr of c-Myc activation. p-value****<0.0001, ***=0.0002, **=0.0062 calculated with Student's t-test. n=4 experiments. Source data are provided as a Source Data file

g) Western blot of the indicated c-Myc targets at 24 and 48 hr of c-Myc activation. GAPDH is a loading control. Representative of n=3 experiments.

h) Cell cycle distribution determined by EdU staining and flow cytometry analysis of c-Myc ER cells with and without 4OH-T addition at the indicated time points. p-value calculated with two-sided Student's t-test. n=3 experiments. Mean with SD is shown. Source data are provided as a Source Data file

i) Western blot of γH2AX at the indicated time-points upon 4OH-T addition in RPE1 c-Myc ER and RPE1 ER empty cell lines. GAPDH is a loading control. Representative of n=3 experiments.

j) Western blot of the indicated proteins at 24 and 48 hr of c-Myc activation. Vinculin is a loading control. Representative of n=3 experiments.

k) Histograms reporting the distribution of fibre length for control and c-Myc-induced cells at the indicated times of c-Myc activation in RPE1 c-Myc ER and RPE1 ER empty cell lines; RPE1 c-Myc ER: p-value****<0.0001 calculated with Mann-Whitney test. RPE1 ER empty: p-value*=0.01 calculated with two-sided Mann-Whitney test. Pool of n=3 experiments. Source data are provided as a Source Data file

l) Cell cycle profile at the indicated time-points after release from G1 with and without c-Myc activation. Early: between 16 and 18 hr; middle: between 20 and 22 hr; late: 24 hr.

m) Repeat of Fig. 1d. Histograms reporting the distribution of fibre length for control and c-Myc-induced cells at times after release from arrest; early=18 hr, middle=22 hr, late=24 hr; p-value****<0.0001 calculated with two-sided Mann-Whitney test. Repeat 2 of n=2 experiments. Source data are provided as a Source Data file

n) Schematic of the synchronisation experiments for G1 release with nocodazole. RPE1 c-Myc ER cells were treated with nocodazole for 8 hr. After mitotic shake-off cells were plated in fresh media. After cells were released into G1, 4OH-T was added to induce c-Myc or left untreated as control.

o) Representative images of RPA and γH2AX immunofluorescence after 20h release from nocodazole arrest.

p) Immunofluorescence staining of chromatin-bound RPA and γH2AX after 20h release from nocodazole arrest. Scatter plot showing the intensity of RPA and γH2AX signal in single nuclei. Black=RPA negative cells, orange=RPA positive cells, red=RPA positive cells with higher γH2AX signal. n=1 experiment. Source data are provided as a Source Data file

q) Graph showing γH2AX intensity in individual S phase cells after 20h release from nocodazole arrest plotted in the scatter plot. p-value****<0.0001 calculated with two-sided Mann-Whitney test. n=1 experiment. Mean with SD is shown. Source data are provided as a Source Data file

r) Western blot of γH2AX at the indicated time-points after release from nocodazole arrest, with and without c-Myc activation. GAPDH is a loading control. Representative of n=2 experiments.

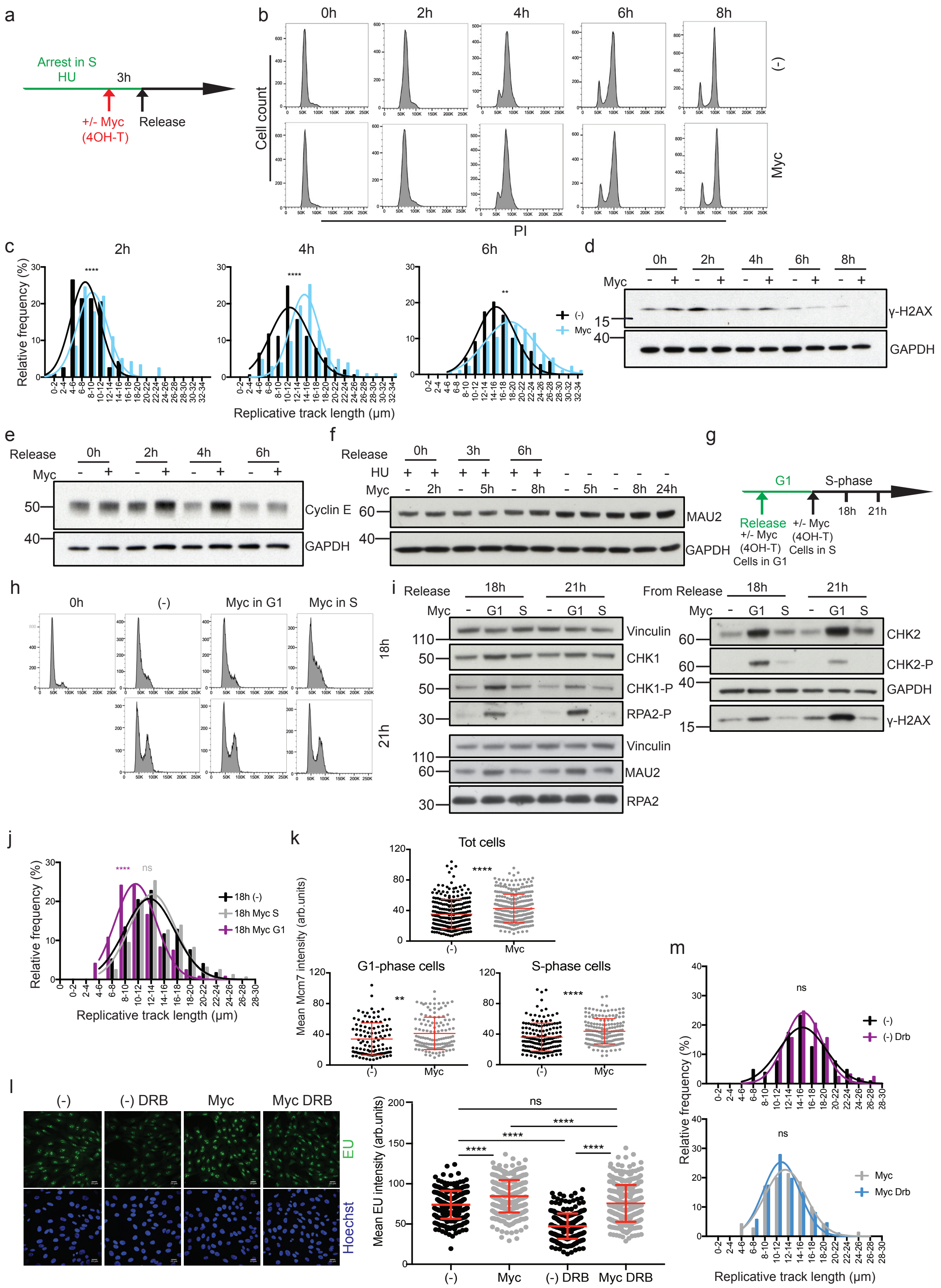

Supplementary figure 2. Replication initiation and replication-transcription collisions are not involved in generating replication stress and DNA damage upon c-Myc activation.

a) Schematic of the synchronisation experiments for S release with HU. RPE1 c-Myc ER cells were arrested in HU overnight. 4-OHT was added 3 hr before release. Cells were then washed and released in S.
b) Cell cycle profile at the indicated time-points after release from S with and without c-Myc activation.
c) Histograms reporting the distribution of fibre length for control and c-Myc-induced cells at the reported times after release from HU arrest; p-value****<0.0001, **=0.006 calculated with two-sided Mann-Whitney test. n=1 experiment. Source data are provided as a Source Data file
d) Western blot of γH2AX at the indicated time-points after release from HU-arrest, with and without c-Myc activation. GAPDH is a loading control. Representative of n=3 experiments.
e) Western blot of Cyclin E at the indicated time-points after release from HU-arrest, with and without c-Myc activation. GAPDH is a loading control. Representative of n=3 experiments.
f) Western blot of MAU2 at the indicated time-points after release from HU-arrest and in asynchrounous cells, with and without c-Myc activation for the indicated time-points. GAPDH is a loading control. Representative of n=3 experiments.
g) Schematic of the synchronisation experiments for G1 release with Palbociclib. RPE1 c-Myc ER cells were arrested in G1 for 24 hr. Cells were then released and 4OH-T was added immediately upon release or 14 hr later, when cells enter S-phase. Cells were then collected at 18 and 21 hr after release.
h) Cell cycle profile at the indicated time-points after release from Palbociclib G1-arrest with and without c-Myc activation in G1 phase, for 18 hr or 21 hr immediately after release, or when cells are in S phase, for 4 hr or 7 hr respectively.
i) Western blot of the indicated proteins after release from Palbociclib G1-arrest, with and without c-Myc activation when cells are in G1 phase, for 18 hr or 21 hr immediately after release, or when cells are in S phase, for 4 hr or 7 hr respectively. GAPDH and Vinculin are loading controls. Representative of n=3 experiments.
j) Histograms reporting the distribution of fibre length for control and c-Myc-induced cells at the reported times after release from Palbociclib G1-arrest in G1 phase, for 18 hr immediately after release, or when cells are in S phase, for 4 hr.; p-value****<0.0001 calculated with two-sided Mann-Whitney test. n=1 experiment. Source data are provided as a Source Data file
k) Graph showing chromatin-bound Mcm7 intensity in individual total, G1 and S phase pre-extracted nuclei after 18 hr from release from G1, plotted in the scatter plot. p-value****<0.0001 calculated with the two-sided Mann-Whitney test. n=1 experiment. Mean with SD is shown. Source data are provided as a Source Data file
l) Left: representative images of EU levels in control and c-Myc-induced cells with and without the addition of DRB for 2 hr. Right: graph showing EU intensity in individual cell nuclei plotted in the scatter plot at 20 hr after release from G1-arrest with and without the addition of DRB for 2 hr and c-Myc activation; p-value****<0.0001 calculated with two-sided Mann-Whitney test. Representative of n=3 experiments. Mean with SD is shown. Source data are provided as a Source Data file
m) Histograms reporting the distribution of fibre length for control and c-Myc-induced cells at 20 hr after release from G1-arrest with and without the addition of DRB for 2 hr; p-value calculated with two-sided Mann-Whitney test. Representative of n=3 experiments. Source data are provided as a Source Data file

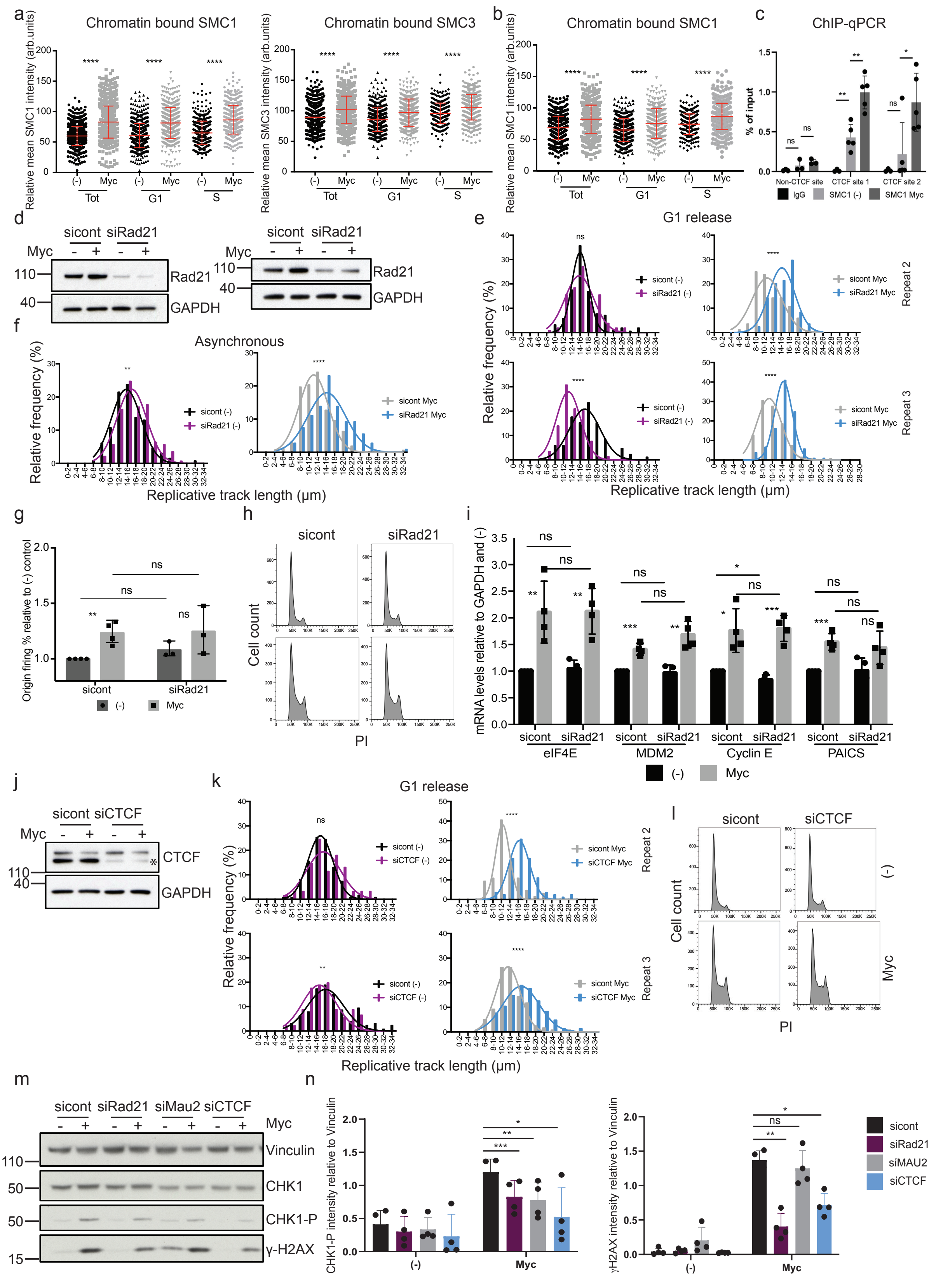

Supplementary figure 3. Reducing the levels of cohesin on chromatin or at CTCF sites prevents c-Myc induced replication stress without affecting the extent of origin firing and the cell cycle profiles.

a) Synchronised cells were released into the cell cycle and immunofluorescence of chromatin-bound cohesin subunits SMC1 and SMC3 were performed at 14 hr after release. Graphs reporting the intensity of SMC1 and SMC3 chromatin signals in total, S phase and G1 phase single nuclei of untreated and c-Myc cells. p-value ****<0.0001 calculated with two-sided Mann-Whitney test. Pool of n=3 experiments. Mean with SD is shown. Source data are provided as a Source Data file

b) Graphs reporting the intensity of SMC1 chromatin signals in total, S phase and G1 phase single nuclei in asynchronous population at 16 hr of c-Myc activation and in untreated cells; p-value****<0.0001 calculated with two-sided Mann-Whitney test. Pool of n=3 experiments. Mean with SD is shown. Source data are provided as a Source Data file

c) Graph representing the ChIP qPCR with the indicated antibodies, at two reported CTCF sites or a reported non-CTCF site as negative control. Mean and standard deviation of at least 3 biological repeats. p-value**=0.0013, 0.0051 , *=0.0266 calculated with two-sided Student's t-test. n=3 or 5 experiments. Mean with SD is shown. Source data are provided as a Source Data file

d) Western blot showing Rad21 knock down in synchronous (left) and asynchronous (right) cells. GAPDH is a loading control.

e) Histograms reporting the distribution of fibre length in synchronised sicontrol and siRad21 depleted cells. p-value****<0.0001 calculated with two-sided Mann-Whitney test. Repeats 2 and 3, Repeat 1 is in fig. 3. Source data are provided as a Source Data file

f) Histograms reporting the distribution of fibre length in asynchronous sicontrol and siRad21 depleted cells. p-value****<0.0001, **=0.0026 calculated with two-sided Mann-Whitney test. Representative of n=3 experiments. Source data are provided as a Source Data file

g) Percentages of origin firing at 20 hr after release from G1-arrest and Rad21 or sicontrol knock down, with and without c-Myc activation. p-value**=0.0035 calculated with two-sided Student's t-test. n=3 experiments. Mean with SD is shown. Source data are provided as a Source Data file

h) Cell cycle profile at 20 hr after release from G1 and Rad21 or sicontrol knock down, with and without c-Myc activation. Representative of n=3 experiments.

i) mRNA levels of different c-Myc targets in control and Rad21 depleted cells at 24 hr of c-Myc activation. p-value calculated with two-sided Student's t-test. n=4 experiments. Mean with SD is shown. Source data are provided as a Source Data file

j) Western blot showing CTCF knock down in synchronous cells. GAPDH is a loading control.

k) Histograms reporting the distribution of fibre length in synchronised sicontrol and siCTCF depleted cells. p-value****<0.0001, **=0.0078 calculated with two-sided Mann-Whitney test. Repeats 2 and 3, repeat 1 is in fig. 3. Source data are provided as a Source Data file

l) Cell cycle profile at 20 hr after release from G1 and CTCF or sicontrol knock down, with and without c-Myc activation. Representative of n=3 experiments.

m) Western blot of the indicated proteins in control, Rad21, CTCF and MAU2 depleted cells upon 24 hr of c-Myc activation. Vinculin is a loading control. Representative of n=4 experiments.

n) Western blot quantification of the indicated proteins in control, Rad21, CTCF and MAU2 depleted cells upon 24 hr of c-Myc activation relative to Vinculin. p-value calculated with two-sided Student's t-test. Left panel p-value***=0.0008, p-value**=0.0049, p-value*=0.026. Right panel p-value**=0.0082, p-value*=0.019. n=4 experiments. Mean with SD is shown. Source data are provided as a Source Data file

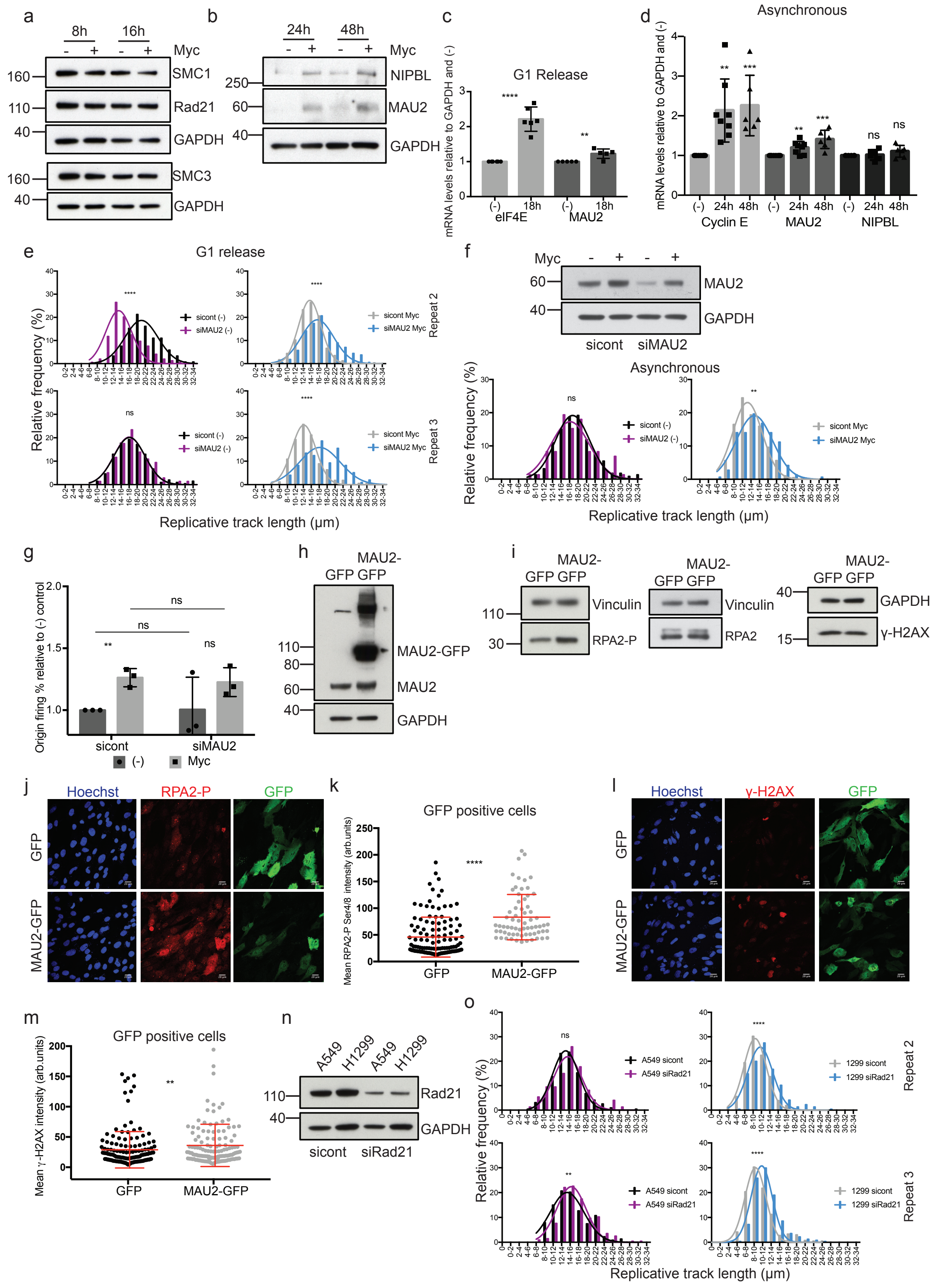

Supplementary figure 4. Dysregulation of the cohesin loader MAU2 is required for c-Myc-induced replication stress.

a) Western blot showing total levels of cohesin subunits and regulators in asynchronous population with and without c-Myc activation for the indicated time-points. Representative experiment of n=2.

b) Western blot showing total levels of the cohesin loaders MAU2 and NIPBL in asynchronous cells at 24 and 48 hr of c-Myc activation. Representative experiment of n=3.

c) mRNA levels of MAU2 at 18 hr of c-Myc activation in synchronised cells. p-value****<0.0001, **=0.0056 calculated with two-sided Student's t-test. n=6 experiments. Mean with SD is shown. Source data are provided as a Source Data file

d) mRNA levels of MAU2 and NIPBL at the indicated time-points of c-Myc activation in asynchronous population. p-value***= 0.0005, 0.0003, **= 0.0012, 0.0042 calculated with two-sided Student's t-test. n=6 experiments. Mean with SD is shown. Source data are provided as a Source Data file

e) Histograms reporting the distribution of fibre length in synchronised sicontrol and siMAU2 depleted cells. p-value****<0.0001 calculated with two-sided Mann-Whitney test. Repeats 2 and 3, repeat 1 is in fig. 4. Source data are provided as a Source Data file

f) Top: Western blot showing MAU2 knock-down in asynchronous population. GAPDH is a loading control. Bottom: histograms reporting the distribution of fibre length in asynchronous sicontrol and siMAU2 depleted cells. p-value**=0.0057 calculated with two-sided Mann-Whitney test. n=1 experiment. Source data are provided as a Source Data file

g) Percentages of origin firing at 20 hr after release from G1-arrest and MAU2 knock-down, with and without c-Myc activation. p-value**=0.0034 calculated with two-sided Student's t-test. n=3 experiments. Mean with SD is shown. Source data are provided as a Source Data file

h) Western blot showing total levels of MAU2 in RPE1 h-TERT cells after transient transfection with GFP and MAU2-GFP plasmids for 24 hr. Representative of n=4 experiments.

i) Western blot showing total levels of the indicated proteins in RPE1 h-TERT cells after transient transfection with GFP and MAU2-GFP plasmids for 24 hr. Vinculin and GAPDH are loading controls. Representative of n=4 experiments.

j) Representative images of GFP and RPA2 phospho S4/8 levels after transient transfection with GFP and MAU2-GFP plasmids for 24 hr.

k) Graph showing RPA2 phospho S4/8 intensity in individual cell nuclei after transient transfection with GFP and MAU2-GFP plasmids for 24 hr plotted in the scatter plot. Only GFP positive cells are shown. p-value****<0.0001 calculated with two-sided Mann-Whitney test. Representative of n=3 experiments. Mean with SD is shown. Source data are provided as a Source Data file

l) Representative images of GFP and γH2AX levels after transient transfection with GFP and MAU2-GFP plasmids for 24 hr.

m) Graph showing γH2AX intensity in individual cell nuclei after transient transfection with GFP and MAU2-GFP plasmids for 24 hr plotted in the scatter plot. Only GFP positive cells are shown. p-value**=0.0074 calculated with two-sided Mann-Whitney test. Representative of n=3 experiments. Mean with SD is shown. Source data are provided as a Source Data file

n) Western blot showing Rad21 knock down in A549 and H1299 cells. GAPDH is a loading control.

o) Histograms reporting the distribution of fibre length in sicontrol and siRad21 depleted A549 and H1299 cells. p-value****<0.0001, **=0.0031 calculated with two-sided Mann-Whitney test. Repeats 2 and 3, repeat 1 is in fig 4. Source data are provided as a Source Data file

METHODS REFERENCES

1 Bertoli, C., Herlihy, A. E., Pennycook, B. R., Kriston-Vizi, J. & de Bruin, R. A. M. Sustained E2F-Dependent Transcription Is a Key Mechanism to Prevent Replication-Stress-Induced DNA Damage. *Cell Rep* **15**, 1412-1422, doi:10.1016/j.celrep.2016.04.036 (2016).

2 Watrin, E. *et al.* Human Scc4 is required for cohesin binding to chromatin, sister-chromatid cohesion, and mitotic progression. *Curr Biol* **16**, 863-874, doi:10.1016/j.cub.2006.03.049 (2006).

3 Petermann, E., Woodcock, M. & Helleday, T. Chk1 promotes replication fork progression by controlling replication initiation. *Proc Natl Acad Sci U S A* **107**, 16090-16095, doi:10.1073/pnas.1005031107 (2010).

4 Di Tommaso, P. *et al.* Nextflow enables reproducible computational workflows. *Nat Biotechnol* **35**, 316-319, doi:10.1038/nbt.3820 (2017).

5 Kurtzer, G. M., Sochat, V. & Bauer, M. W. Singularity: Scientific containers for mobility of compute. *PLoS One* **12**, e0177459, doi:10.1371/journal.pone.0177459 (2017).

6 Li, H. & Durbin, R. Fast and accurate short read alignment with Burrows-Wheeler transform. *Bioinformatics* **25**, 1754-1760, doi:10.1093/bioinformatics/btp324 (2009).

7 Li, H. *et al.* The Sequence Alignment/Map format and SAMtools. *Bioinformatics* **25**, 2078-2079, doi:10.1093/bioinformatics/btp352 (2009).

8 Quinlan, A. R. & Hall, I. M. BEDTools: a flexible suite of utilities for comparing genomic features. *Bioinformatics* **26**, 841-842, doi:10.1093/bioinformatics/btq033 (2010).

9 Barnett, D. W., Garrison, E. K., Quinlan, A. R., Stromberg, M. P. & Marth, G. T. BamTools: a C++ API and toolkit for analyzing and managing BAM files. *Bioinformatics* **27**, 1691-1692, doi:10.1093/bioinformatics/btr174 (2011).

10 Kent, W. J., Zweig, A. S., Barber, G., Hinrichs, A. S. & Karolchik, D. BigWig and BigBed: enabling browsing of large distributed datasets. *Bioinformatics* **26**, 2204-2207, doi:10.1093/bioinformatics/btq351 (2010).

11 Zhang, Y. *et al.* Model-based analysis of ChIP-Seq (MACS). *Genome Biol* **9**, R137, doi:10.1186/gb-2008-9-9-r137 (2008).

12 Heinz, S. *et al.* Simple combinations of lineage-determining transcription factors prime cis-regulatory elements required for macrophage and B cell identities. *Mol Cell* **38**, 576-589, doi:10.1016/j.molcel.2010.05.004 (2010).

13 Liao, Y., Smyth, G. K. & Shi, W. featureCounts: an efficient general purpose program for assigning sequence reads to genomic features. *Bioinformatics* **30**, 923-930, doi:10.1093/bioinformatics/btt656 (2014).

14 team, R. c. R: A language and environment for statistical computing. *R Foundation for Statistical Computing* (2017).

15 Love, M. I., Huber, W. & Anders, S. Moderated estimation of fold change and dispersion for RNA-seq data with DESeq2. *Genome Biol* **15**, 550, doi:10.1186/s13059-014-0550-8 (2014).

16 Ewels, P., Magnusson, M., Lundin, S. & Kaller, M. MultiQC: summarize analysis results for multiple tools and samples in a single report. *Bioinformatics* **32**, 3047-3048, doi:10.1093/bioinformatics/btw354 (2016).

17 Daley, T. & Smith, A. D. Predicting the molecular complexity of sequencing libraries. *Nat Methods* **10**, 325-327, doi:10.1038/nmeth.2375 (2013).

18 Ramirez, F. *et al.* deepTools2: a next generation web server for deep-sequencing data analysis. *Nucleic Acids Res* **44**, W160-165, doi:10.1093/nar/gkw257 (2016).
19 Landt, S. G. *et al.* ChIP-seq guidelines and practices of the ENCODE and modENCODE consortia. *Genome Res* **22**, 1813-1831, doi:10.1101/gr.136184.111 (2012).
20 Karolchik, D. *et al.* The UCSC Table Browser data retrieval tool. *Nucleic Acids Res* **32**, D493-496, doi:10.1093/nar/gkh103 (2004).
21 Robinson, J. T. *et al.* Integrative genomics viewer. *Nat Biotechnol* **29**, 24-26, doi:10.1038/nbt.1754 (2011).
22 Wendt, K. S. *et al.* Cohesin mediates transcriptional insulation by CCCTC-binding factor. *Nature* **451**, 796-801, doi:10.1038/nature06634 (2008).
