# Supplementary material for: Oncogenic c-Myc induces replication stress by increasing cohesins chromatin occupancy in a CTCF-dependent manner
Source: Nat Commun. 2024 Feb 21;15:1579. doi: 10.1038/s41467-024-45955-z (PMC10881979; doi:10.1038/s41467-024-45955-z)
Supplement: Supplementary file 3 — Description of Additional Supplementary Files [file 41467_2024_45955_MOESM3_ESM.pdf]

Description of Additional Supplementary Files

File Name: Supplementary Data 1

Description: Primer sequences for RTqPCR
