# Supplementary material for: Oncogenic c-Myc induces replication stress by increasing cohesins chromatin occupancy in a CTCF-dependent manner
Source: Nat Commun. 2024 Feb 21;15:1579. doi: 10.1038/s41467-024-45955-z (PMC10881979; doi:10.1038/s41467-024-45955-z)
Supplement: Supplementary file 5 — Reporting Summary [file 41467_2024_45955_MOESM5_ESM.pdf]

nature portfolio

Corresponding author(s): Rob de Bruin, Cosetta Bertoli

Last updated by author(s): Nov 28, 2023

Reporting Summary

Nature Portfolio wishes to improve the reproducibility of the work that we publish. This form provides structure for consistency and transparency in reporting. For further information on Nature Portfolio policies, see our Editorial Policies and the Editorial Policy Checklist.

Statistics

For all statistical analyses, confirm that the following items are present in the figure legend, table legend, main text, or Methods section.

n/a	Confirmed	
☐	☒	The exact sample size (*n*) for each experimental group/condition, given as a discrete number and unit of measurement
☐	☒	A statement on whether measurements were taken from distinct samples or whether the same sample was measured repeatedly
☐	☒	The statistical test(s) used AND whether they are one- or two-sided<br>*Only common tests should be described solely by name; describe more complex techniques in the Methods section.*
☒	☐	A description of all covariates tested
☐	☒	A description of any assumptions or corrections, such as tests of normality and adjustment for multiple comparisons
☐	☒	A full description of the statistical parameters including central tendency (e.g. means) or other basic estimates (e.g. regression coefficient) AND variation (e.g. standard deviation) or associated estimates of uncertainty (e.g. confidence intervals)
☐	☒	For null hypothesis testing, the test statistic (e.g. *F*, *t*, *r*) with confidence intervals, effect sizes, degrees of freedom and *P* value noted<br>*Give P values as exact values whenever suitable.*
☒	☐	For Bayesian analysis, information on the choice of priors and Markov chain Monte Carlo settings
☒	☐	For hierarchical and complex designs, identification of the appropriate level for tests and full reporting of outcomes
☒	☐	Estimates of effect sizes (e.g. Cohen's *d*, Pearson's *r*), indicating how they were calculated

Our web collection on statistics for biologists contains articles on many of the points above.

Software and code

Policy information about availability of computer code

Data collection	Immunofluorescence images acquired using LASAF software, Flow cytometry data acquired using DIVA software (BD)
Data analysis	Immunofluorescence images were analysed using ImageJ v1.52i and Flow cytometry data using FlowJo v10.0.8. Prism 8 for data display and statistical analysis

For manuscripts utilizing custom algorithms or software that are central to the research but not yet described in published literature, software must be made available to editors and reviewers. We strongly encourage code deposition in a community repository (e.g. GitHub). See the Nature Portfolio guidelines for submitting code & software for further information.

Data

Policy information about availability of data

All manuscripts must include a data availability statement. This statement should provide the following information, where applicable:

- Accession codes, unique identifiers, or web links for publicly available datasets
- A description of any restrictions on data availability
- For clinical datasets or third party data, please ensure that the statement adheres to our policy

Sequencing data have been deposited in GEO under accession code GSE146766

Research involving human participants, their data, or biological material

Policy information about studies with human participants or human data. See also policy information about sex, gender (identity/presentation), and sexual orientation and race, ethnicity and racism.

Reporting on sex and gender	*Use the terms sex (biological attribute) and gender (shaped by social and cultural circumstances) carefully in order to avoid confusing both terms. Indicate if findings apply to only one sex or gender; describe whether sex and gender were considered in study design; whether sex and/or gender was determined based on self-reporting or assigned and methods used. Provide in the source data disaggregated sex and gender data, where this information has been collected, and if consent has been obtained for sharing of individual-level data; provide overall numbers in this Reporting Summary. Please state if this information has not been collected. Report sex- and gender-based analyses where performed, justify reasons for lack of sex- and gender-based analysis.*
Reporting on race, ethnicity, or other socially relevant groupings	*Please specify the socially constructed or socially relevant categorization variable(s) used in your manuscript and explain why they were used. Please note that such variables should not be used as proxies for other socially constructed/relevant variables (for example, race or ethnicity should not be used as a proxy for socioeconomic status). Provide clear definitions of the relevant terms used, how they were provided (by the participants/respondents, the researchers, or third parties), and the method(s) used to classify people into the different categories (e.g. self-report, census or administrative data, social media data, etc.) Please provide details about how you controlled for confounding variables in your analyses.*
Population characteristics	*Describe the covariate-relevant population characteristics of the human research participants (e.g. age, genotypic information, past and current diagnosis and treatment categories). If you filled out the behavioural & social sciences study design questions and have nothing to add here, write "See above."*
Recruitment	*Describe how participants were recruited. Outline any potential self-selection bias or other biases that may be present and how these are likely to impact results.*
Ethics oversight	*Identify the organization(s) that approved the study protocol.*

Note that full information on the approval of the study protocol must also be provided in the manuscript.

Field-specific reporting

Please select the one below that is the best fit for your research. If you are not sure, read the appropriate sections before making your selection.

☒ Life sciences ☐ Behavioural & social sciences ☐ Ecological, evolutionary & environmental sciences

For a reference copy of the document with all sections, see nature.com/documents/nr-reporting-summary-flat.pdf

Life sciences study design

All studies must disclose on these points even when the disclosure is negative.

Sample size	For DNA fibre analysis sample size was determined using published results Techer et al 2013. Other sample sizes were based on previously reported data
Data exclusions	Flow cytometry data was excluded based on the gating strategy described. Otherwise there were no data exclusions
Replication	Fig 1d,f,g, 2c,d, e, f 4b, e n=2 experimental repeats, Fig 1h, 2a, b, 3b, d, f, h, 4a, c, g, h, i, k n=3 experimental repeats. Sup fig 1c, g, h, i, j, k, 2d, e, f, i, l, m, 3a, b, c, e, f, g, h, k, l, 4b, e, g, k, m, o n=3 experimental repeats, Sup fig 1m, r, 4a n=2. Sup fig 1d, e, f, 3i, m, n, 4h, i n=4. Suppl fig 1b, p, q, 2c, j, k, 4f n=1. Sup fig 4d n=6.
Randomization	*Describe how samples/organisms/participants were allocated into experimental groups. If allocation was not random, describe how covariates were controlled OR if this is not relevant to your study, explain why.*
Blinding	*Describe whether the investigators were blinded to group allocation during data collection and/or analysis. If blinding was not possible, describe why OR explain why blinding was not relevant to your study.*

Reporting for specific materials, systems and methods

We require information from authors about some types of materials, experimental systems and methods used in many studies. Here, indicate whether each material, system or method listed is relevant to your study. If you are not sure if a list item applies to your research, read the appropriate section before selecting a response.

Materials & experimental systems

n/a	Involved in the study
☐	☒ Antibodies
☐	☒ Eukaryotic cell lines
☒	☐ Palaeontology and archaeology
☒	☐ Animals and other organisms
☒	☐ Clinical data
☒	☐ Dual use research of concern
☒	☐ Plants

Methods

n/a	Involved in the study
☐	☒ ChIP-seq
☐	☒ Flow cytometry
☒	☐ MRI-based neuroimaging

Antibodies

Antibodies used: anti-Rpa2 (Millipore RPA34-20), anti-Phospho-Histone H2A.X (γH2AX) (Ser139) (Cell Signaling Technology g-H2AX 20E3), anti-Smc1 (Bethyl laboratories A300-055A), anti-Smc3 (Bethyl laboratories A300-060A) and anti-Mcm7 (Santa Cruz Biotechnology sc-56324) , anti-Scc4 (Mau2) (Abcam ab183033), anti -Idn3 (Nipbl) (Abcam ab106768), anti-Rad21 (Abcam ab992), anti-CyclinE (Santa Cruz Biotechnology HE12 sc-247), anti-CTCF (Chip-grade AB70303 Abcam), anti-RPA32 Phospho S4/S8 (Bethyl laboratories A300-245A) and anti-GFP (Abcam AB1218), anti-c-Myc (Santa Cruz Biotechnology 9E10 sc-40), anti-CHK1 (Cell Signalling Technology 2360), anti CHK1 Phospho S345 (Cell Signalling Technology 2341), anti-CHK2 (Cell Signalling Technology 2662), anti CHK2 Phospho Thr68 (Cell Signalling Technology 2661), anti-p21 (Cell Signalling Technology 2947), anti-MDM2 (Cell Signalling Technology 86934).

Validation: The antibodies are commercially available and already validated in the field. The use of positive and negative controls in the experiments is used to assess their reliability.

Eukaryotic cell lines

Policy information about cell lines and Sex and Gender in Research

Cell line source(s): Retinal Pigment Epithelia 1 (ATCC CRL-4000) c-Myc-ER cells were published in Bertoli, Herlihy et al, 2016. RPE1-ER empty cells were generated in the lab as described in Bertoli, Herlihy et al, 2016. RPE1 h-TERT (ATCC CRL-4000), A549 (ATCC CCL-185), H1299 (ATCC CRL-5803).

Authentication: Cell lines were not authenticated

Mycoplasma contamination: All cell lines tested negative for mycoplasma contamination

Commonly misidentified lines (See ICLAC register): *Name any commonly misidentified cell lines used in the study and provide a rationale for their use.*

Plants

Seed stocks: *Report on the source of all seed stocks or other plant material used. If applicable, state the seed stock centre and catalogue number. If plant specimens were collected from the field, describe the collection location, date and sampling procedures.*

Novel plant genotypes: *Describe the methods by which all novel plant genotypes were produced. This includes those generated by transgenic approaches, gene editing, chemical/radiation-based mutagenesis and hybridization. For transgenic lines, describe the transformation method, the number of independent lines analyzed and the generation upon which experiments were performed. For gene-edited lines, describe the editor used, the endogenous sequence targeted for editing, the targeting guide RNA sequence (if applicable) and how the editor was applied.*

Authentication: *Describe any authentication procedures for each seed stock used or novel genotype generated. Describe any experiments used to assess the effect of a mutation and, where applicable, how potential secondary effects (e.g. second site T-DNA insertions, mosiacism, off-target gene editing) were examined.*

ChIP-seq

Data deposition

☒ Confirm that both raw and final processed data have been deposited in a public database such as GEO.

☐ Confirm that you have deposited or provided access to graph files (e.g. BED files) for the called peaks.

Data access links (*May remain private before publication.*): GEO accession code GSE146766 and GSE249375

Files in database submission: fastq, bigWig, narrowPeak.

Genome browser session (e.g. UCSC) | No longer applicable

Methodology

Replicates	Two independet biological replicates per condition were analyzed (for both GSE)
Sequencing depth	20 million each sample (for both GSE)
Antibodies	anti-Smc1 (Bethyl laboratories A300-055A), Santa xcruz mouse IgG (sc-2025)
Peak calling parameters	The nf-core/chipseq pipeline (version 1.0.0, https://doi.org/10.1101/610741; https://doi.org/10.5281/zenodo.3240507) written in the Nextflow domain specific language (version 0.32.0; Di Tommaso et al., 2017) was used to perform the primary analysis of the samples in conjunction with Singularity (version 2.6.0; Kurtzer et al., 2017).The command used was "nextflow run nf-core/chipseq --design design.csv --genome hg19 --singleEnd --narrowPeak --min_reps_consensus 2 -profile crick -r 1.0.0".All data was processed relative to the human UCSC hg19 genome (Karolchik et al., 2004) downloaded from AWS iGenomes (https://github.com/ewels/AWS-iGenomes). Gene annotation files in GTF format were originally downloaded from UCSC on July 17th 2015.
Data quality	The DNA library for sequencing was prepared using 2 ng of ChIP'ed DNA for the Kapa Hyper Prep (GSE14766) or Ovation Ultralow V2 TECAN (GSE249375) kit according to the manufacturer instructions. QC was performed using the ThermoFisher Qubit and either the Agillent BioAnalyser or TapeStation. The DNA samples were normalised and prepared into Illumina compatible libraries using the KAPA HyperPrep kit according to the manufacturer's instructions. The libraries were pooled to 4 nM and sequencing was performed on the HiSeq 4000 with at least 75 bp reads.<br>GSE249375 samples were spiked in with Drosophila chromatin and analyzed according to manufacture's instructions (Active Motif).
Software	For a condensed overview of the pipeline, the bioinformatics tools used in each step and an extensive list of citations please see the pipeline homepage (https://github.com/nf-core/chipseq).

Flow Cytometry

Plots

Confirm that:

- [ ] The axis labels state the marker and fluorochrome used (e.g. CD4-FITC).
- [x] The axis scales are clearly visible. Include numbers along axes only for bottom left plot of group (a 'group' is an analysis of identical markers).
- [ ] All plots are contour plots with outliers or pseudocolor plots.
- [ ] A numerical value for number of cells or percentage (with statistics) is provided.

Methodology

Sample preparation	*Describe the sample preparation, detailing the biological source of the cells and any tissue processing steps used.*
Instrument	BD LSRII
Software	DIVA Software (BD) used to collect the data and FlowJo software used for analysis
Cell population abundance	No FACS sorting was completed, so no populations selected.
Gating strategy	Live cells were gated using forward and side scatter. Single cells were gated according to peak area and height. Populations in Fig suplementary 1h, were defined as follows: G1: EdU negative and G1 DNA content, S: EdU positive, G2: EdU negative and G2 DNA content.

- [ ] Tick this box to confirm that a figure exemplifying the gating strategy is provided in the Supplementary Information.
